# Gender differences in predictors of self-rated health among older adults in Brazil and Chile

**DOI:** 10.1186/s12889-015-1666-9

**Published:** 2015-04-11

**Authors:** Ana Cristina Viana Campos, Cecilia Albala, Lydia Lera, Hugo Sánchez, Andréa Maria Duarte Vargas, Efigênia Ferreira e Ferreira

**Affiliations:** School of Dentistry, Universidade Federal de Minas Gerais, Presidente Antônio Carlos 6627, Belo Horizonte, Minas Gerais 31270-901 Brazil; Unidad Nutrición, Salud Pública y Envejecimiento Saludable, INTA, Universidad de Chile, El Líbano 5524 Macul, Santiago, 138-11 Chile; Department of Community and Preventive Dentistry, School of Dentistry, Universidade Federal de Minas Gerais, Presidente Antônio Carlos 6627, Belo Horizonte, Minas Gerais 31270-901 Brazil

**Keywords:** Self-rated health, Older adults, Gender, Latin American

## Abstract

**Background:**

The determinants of self-rated health (SRH) have been widely investigated to explain social differences and gender differences in health. This study aimed to investigate the gender differences in predictors of SRH among Brazilian and Chilean older adults.

**Methods:**

We used two samples of older people: 2052 Brazilian community-dwelling participants (1226 women and 862 men) and 1301 Chilean community-dwelling participants (855 women and 446 men). Sequential logistic regression analysis was used to examine the relationships between SRH and potential predictors in a hierarchical model.

**Results:**

Overall, 35.5% and 52.1% of individuals in Chile and Brazil, respectively, reported good SRH. There was a gradient association between good SRH and chronic diseases in both countries. Chilean men without chronic disease or with one had a higher chance of good SRH, compared to two or more diseases. For Brazilian men, no or one chronic disease was associated with good SRH. For women, the set of independent predictors for good SRH included no chronic diseases or one chronic disease, and no activities of daily living limitation. For men, the set also included instrumental activities limitation. For Brazilian adults of both genders, depression demonstrated the strongest independent association with good SRH.

**Conclusions:**

We conclude that when examining gender differences in predictors of SRH, the similarities are greater than the differences between Brazilian and Chilean older adults. In both countries, physical health was the most important predictor of SRH. In addition, absence of depression was the strongest predictor of good health in older Brazilian adults.

**Electronic supplementary material:**

The online version of this article (doi:10.1186/s12889-015-1666-9) contains supplementary material, which is available to authorized users.

## Background

In recent decades, Latin American countries have experienced a rapid process of demographic and epidemiological transitions leading to a rapidly ageing population [1]. In the last half century, the percentage of persons aged 60 years and over rose from 6% to 8%. In 2025, it is expected to swell to 56 million, bringing the total older population to 96 million. However, this trend is homogeneous neither at the international nor the national level [2]. In Chile, 11.5% of the population is older than 60 years and is expected to reach about 20.1% by 2025 [3]. In 2010–2015, the life expectancy at birth, currently the highest in Latin America, is estimated at 76.1 years for men and 82.2 years for women [4]. In Brazil, there are approximately 25 million people aged 60 or older (10.8% of the population) [5]. Projections for 2030, estimate a life expectancy in Brazil around 77.4 years. In 2050 almost 30% of the Brazilian population will be 60 years and older, placing Brazil as one of the countries with the largest absolute number of older people worldwide [6].

However, ageing in both countries is occurring even in the context of health inequalities, with high rates of poverty, relative low coverage and quality of health and pension systems [7-10]. Among countries with available data, Brazil is among the countries with the worst income distribution in Latin America (Gini coefficient = 0.547), but Chile was no exception to this situation (Gini coefficient = 0.521) [11]. In addition, Brazilian illiteracy is high (24%) [5] compared to the Chile (12%) [1].

The characterisation of the health conditions of older people requires detailed information about different aspects of life, such as demographic and socioeconomic factors, chronic diseases and functional capacity [8,12]. In addition to acting as a social gradient, the unfavourable economic and social circumstances also affect health over the life span, making the health of older people even more susceptible to social determination by the accumulation of exposures to risk factors [13].

Self-rated health (SRH) is a multidimensional construct, which includes physical, psychological, functional and social variables [14] and has been used in population surveys in several countries [15-17]. The World Health Organization (WHO) recommended this indictor to verify health in population-based studies including older individuals.

In general, SRH among older adults is positive. The multicentre SABE study (Salud, Bienestar y Envejecimiento) [18] was conducted in the following seven cities of Latin America and the Caribbean: Buenos Aires, Argentina; Bridgetown, Barbados; Havana, Cuba; Montevideo, Uruguay; Santiago, Chile; Mexico City, DF, Mexico and São Paulo, Brazil. In São Paulo, 44.2% of women and 48.4% of men reported good/very good SRH. In Santiago, this prevalence was lower for both sexes (33.9% and 44.2%, respectively). In other SABE countries, good SRH ranged from 27.9% among men in Mexico to 69.0% among men in Uruguay [18].

The determinants of SRH have been widely investigated to explain social differences [19] and gender differences in health [20,21]. This study aimed to investigate the gender differences in predictors of SRH among Brazilian and Chilean older people.

## Methods

### Participants

This study concerns the analysis of the baseline cohort AGEQOL study and the Chilean sample of SABE.

“Aging, Gender and Quality of Life (AGEQOL)” is a cohort study in Sete Lagoas, Brazil with a representative sample of 2052 community-dwelling participants (1226 women and 862 men), aged 60 and older. Data collection was conducted between January and July 2012. The sampling process was conducted in two stages: in the first, census tracts were selected and in the second, households within each sector were selected. In each household, all residents aged 60 years or more of both genders, regardless of your marital status or kinship were interviewed. All persons 60+ years in the selected households were informed of the study and were asked to sign an informed consent form that had been previously approved by the Ethical Committee of the Federal University of Minas Gerais. The interviews lasted 40 to 60 minutes. At the end of the interviews, each subject in the city received guidance regarding health care and activity options as well as the personal contact information of the researcher responsible for the questionnaire [22].

The Chilean sample cohort SABE includes a representative sample of 1301 participants (855 women and 446 men) aged 60 and older living in private households in Santiago, Chile (January 2000 to January 2011). The primary sampling unit (PSU) was a conglomerate of independent households within a given geographical area. The PSU were divided in turn into secondary sampling units (SSU), each consisting of a smaller number of independent households. These USM joined in turn by tertiary sampling units (UTM), consisting of selected households that were interviewed all persons aged 60 or older. An informed consent to participate in this study should be obtained from all participants. The protocol for this study has been approved by the Institutional Review Board at Institute of Nutrition and Food Technology (INTA), University of Chile [18].

### Variables

Most variables were dichotomised to enhance the interpretability of the logistic regression coefficients. For ordinal variables, dummy variables were created with the “worst” category used as the referent [23].

The outcome variable, SRH, was assessed with the question: ‘How would you describe your health in the last 30 days?’, with the response options “very good”, “good”, “fair”, “poor” and “very poor” which were dichotomised as poor (fair/poor/very poor) versus good (very good/good).

The socioeconomic and demographic information included age (<75 years, ≥75 years), gender (male, female) marital status (married, separate, widower, single), retirement (yes, no), tertiles of income, years of education (0, 1–4, 5–7, ≥8), and living arrangements (living with spouse, mixed arrangements, living alone). Current smoking, drinking, physical activity and social participation were measured as dichotomous variables (yes, no). The affordability of healthcare was measured by public and others.

Chronic diseases (hypertension, diabetes, cardiovascular diseases, musculoskeletal and respiratory diseases) is one of the indicators used to compare health among older people in different countries because it is an easy to obtain indicator that reflects multiple aspects of health that are difficult to capture using other methods in representative samples of the population [24]. The number of diseases was measured in three categories: 0, 1, ≥ 2.

Functional capacity was evaluated from the participants' responses to six basic activities of daily living (ADL) - eating, dressing and undressing, grooming, walking, getting in and out of bed, bathing and continence [25] and seven instrumental activities (IADL) - using the telephone, travel, shopping, meal preparation, housework, taking medicine and management of finances [26]. The participants were classified as restricted if they had one or more ADL limitation(s) or IADL limitation(s) [8]. To evaluate the cognitive status of older people, we used the Mini - Mental State Examination validated in Brazil [27] and in Chile [28], with a cut-off set at 21/22 points [29]. Depressive symptoms were measured by the Geriatric Depression Scale short version (GDS-15) previously validated in Brazil [30] and in Chile [31] with a cut-off of 5/6 points.

### Statistical analyses

**Figure 1:**
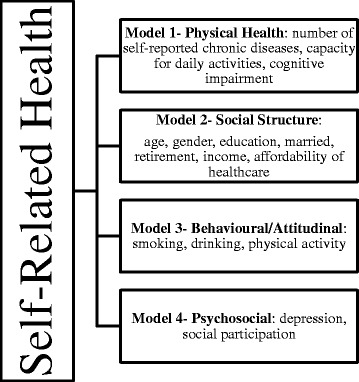
Model predictors of self-rated health (SRH) among older people.

SPSS software (SPSS Institute, Chicago, USA) version 19.0 was used for the analysis including Chi-Square tests and logistic regression. Sequential logistic regression analysis was used to examine the relationships between SRH with potential predictors. The present study used the model from Demirchyan et al. [21] adapted to investigate the factors associated with good perceived health among older people. The independent variables were grouped into conceptually coherent hierarchical blocks: physical health, social structure, behavioural/attitudinal, psychosocial (Figure 1). In model 1, we examined the association between SRH and chronic diseases, ADL limitation, IADL limitation and cognitive impairment (physical health). In addition, we adjusted for variables of social structure (age, sex, marital status, education, income, retired, household arrangement and healthcare) in Model 2. Smoking, drinking and physical activity comprised the third level of adjustment in Model 3 (behavioural/attitudinal). Finally, we adjusted for psychosocial level (depression and social participation) in Model 4. Considering that the sets of determinants of SRH could vary among women and men [20], we performed regression stratified by gender with the variables that remained in the final model.

Odds ratios (OR) and 95% confidence intervals (CI95%) for good SRH were calculated, and p values less than 0.05 (two-tailed test) were considered statistically significant. In addition, homogeneity and multicollinearity tests showed that all assumptions of the model were satisfied.

## Results

### Descriptive characteristics

In Brazilian sample the mean age was 70.9 ± 8.1 years (71.3 ± 8.3 for women and 70.7 ± 7.8 for men). The mean age of the Chilean sample at the start of follow-up was 72 ± 8.2 years (73.0 ± 8.5 women and 71.0 ± 7.5 for men).

Table 1 provides descriptive statistics by gender for both countries. Most men (70.8% for Brazilian and 72.2% for Chilean) and women (68.7% for Brazilian and 62.7% for Chilean) were aged 60–74 years old. Gender differences in ages only emerged among Chilean older people. Taking into account the three groups of living arrangements established in this study, there were 2.5% more women living alone in Brazil (16.6%) and Chile (14.2%) compared to men. For Brazilian older adults, we observed gender differences in living arrangements (p < 0.001).

**Table 1:** **Descriptive characteristics by gender**

**Variables**	**Chile**			**Brazil**		
	**Total**	**Men**	**Women**	**Total**	**Men**	**Women**
	**(N = 1301)**	**(N = 446)**	**(N = 855)**	**(N = 2052)**	**(N = 826)**	**(N = 1226)**
**Physical health**						
**Chronic Diseases**	**n (%)**	**n (%)**	**n (%)**	**n (%)**	**n (%)**	**n (%)**
≥ 2	654 (53)	171 (41.2)	483 (58.8)	1099 (53.6)	368 (44.6)	731 (59.6)
1	452 (36.6)	182 (44)	270 (32.9)	626 (30.5)	274 (33.2)	352 (28.7)
0	129 (10.5)	61 (14.7)	68 (8.3)	327 (15.9)	184 (22.3)	143 (11.7)
**ADL Limitation**						
Yes	251 (19.3)	45 (10.1)	206 (24.1)	160 (7.8)	98 (8.0)	62 (7.5)
No	1044 (80.3)	398 (89.2)	646 (75.6)	1892 (92.2)	764 (92.5)	1128 (92.0)
**IADL Limitation**						
Yes	374 (28.9)	78 (17.6)	296 (34.7)	600 (29.2)	196 (23.7)	404 (33.0)
No	922 (71.1)	365 (82.4)	557 (65.3)	1452 (70.8)	633 (76.3)	822 (67.0)
**Cognitive Impairment**						
Yes	118 (9.1)	30 (6.7)	88 (10.3)	264 (12.9)	98 (11.9)	166 (13.5)
No	1183 (90.9)	416 (93.3)	767 (89.7)	1788 (87.1)	728 (88.1)	1060 (86.5)
**Social structure**						
**Age**						
<75 years old	858 (65.9)	322 (72.2)	536 (62.7)	1427 (69.5)	585 (70.8)	842 (68.7)
≥75 years old	443 (34.1)	124 (27.8)	319 (37.3)	579 (28.2)	241 (29.2)	339 (27.7)
**Marital Status**						
Married	451 (34.7)	234 (52.5)	217 (25.4)	1084 (52.9)	615 (74.5)	469 (38.3)
Separate	249 (19.1)	86 (19.3)	163 (19.1)	154 (7.5)	59 (7.1)	95 (7.8)
Widower	397 (46.4)	107 (24)	504 (38.7)	612 (29.9)	93 (11.3)	519 (42.4)
Single	95 (7.3)	18 (4)	77 (9)	199 (9.7)	59 (7.1)	140 (11.4)
**Years of Education**						
0	222 (17.1)	54 (12.1)	168 (19.7)	579 (28.2)	240 (29.1)	339 (27.7)
1–4	339 (26.1)	111 (24.9)	228 (26.7)	1282 (62.5)	500 (60.5)	782 (63.8)
5–7	365 (28.1)	133 (29.8)	232 (27.1)	130 (6.3)	63 (7.6)	67 (5.5)
>8	375 (28.8)	148 (33.1)	227 (26.6)	61 (3.0)	23 (2.8)	38 (3.1)
**Income**						
1° tertile	361 (30.7)	91 (21.5)	270 (35.9)	1357 (66.1)	480 (58.1)	877 (71.5)
2° tertile	384 (32.7)	125(29.6)	259 (34.4)	505 (24.6)	246 (29.8)	259 (21.1)
3° tertile	430 (36.6)	207 (48.9)	223(29.7)	190 (9.3)	100 (12.1)	90 (7.3)
**Retired**						
Yes	397 (33.5)	160 (36)	237 (31.9)	1518 (74.0)	699 (84.6)	819 (66.8)
No	790 (66.5)	285 (64)	505 (68.1)	534 (26.0)	27 (15.4)	407 (33.2)
**Household Arrangement**						
Living with spouse	284 (21.8)	94 (21.1)	149 (16.3)	1065 (53.0)	616 (75.5)	449 (37.6)
Mixed arrangements	851 (65.4)	307 (68.8)	544(63.6)	668 (33.2)	121 (14.8)	547 (45.8)
Living alone	166 (12.8)	45 (10.1)	121 (14.2)	277 (13.8)	79 (9.7)	198 (16.6)
**Healthcare**						
Public	1089 (83.7)	359 (80.5)	730 (85.4)	1153 (56.2)	477 (57.7)	676 (55.1)
Others	63 (4.8)	25 (5.6)	38 (4.4)	899 (43.8)	349 (42.3)	550 (44.9)
**Behavioural/attitudinal**						
**Currently Smoking**						
Yes	158 (12.2)	78 (17.6)	80 (6.7)	225 (11.0)	143 (17.3)	82 (6.7)
No	1139 (87.8)	365 (82.4)	774 (90.6)	1827 (89.0)	683 (82.7)	1144 (93.3)
**Drinking**						
Yes	92 (7.1)	65 (14.6)	27 (3.2)	383 (18.7)	171 (17.4)	212 (19.8)
No	1206 (92.9)	379 (85.4)	827 (96.8)	1669 (81.3)	569 (68.9)	1100 (89.7)
**Physical Activity**						
Yes	270 (20.8)	121 (27.2)	149 (17.5)	545 (26.6)	216 (26.2)	329 (26.8)
No	1028 (79.2)	324 (72.8)	704 (82.5)	1507 (73.4)	610 (73.8)	897 (73.2)
**Psychosocial**						
**Symptoms of Depression**						
Yes	854 (26.3)	92 (22.7)	213 (28.3)	619 (30.2)	197 (23.8)	422 (34.4)
No	854 (73.7)	314 (77.3)	540 (71.7)	1433 (69.8)	785 (95.0)	804 (65.6)
**Social Participation**						
Yes	408 (31.5)	108 (24.3)	300 (35.2)	157 (7.7)	41 (5.0)	116 (9.5)
No	889 (68.5)	336 (75.7)	553 (64.8)	1895 (92.3)	900 (91.6)	1110 (90.5)

ADL: activities of daily living.

IADL: instrumental activities of daily living.

The percentage of illiteracy among Brazilian older people was higher than among Chilean older people, 28.2% and 17.1% respectively. These percentages were higher among Chilean women (19.7%) and Brazilian men (29.1%). There were gender differences in years of education among Chilean older adults (p = 0.002). In Brazil, only 10.4% of men and 8.6% of women reported over 4 years of study. By contrast, for Chilean older people, those percentages were 62.9% and 53.7%, respectively (Table 1).

There were statistically significant gender differences for both countries related to marital status, income, retirement, current smoking and drinking. The great majority of men in the sample were married (74.5% for Brazilian and 52.5% for Chilean). Most Brazilian older people had low monthly income (66.1%), even higher among females (71.5%) compared to males (58.1%). The income of Chilean older people was more homogeneous (Table 1).

Only 15.9% and 10.5% of the Brazilian and Chilean older people did not have chronic disease, respectively. However, the percentage of women (59.6% for Brazilian and 58.8% for Chilean) with more than two diseases was statistically higher (p < 0.001) compared to men (44.6% for Brazilian and 41.2% for Chilean). The prevalence of cognitive impairment was 12.9% among Brazilian adults and 9.1% among Chilean, with significant gender differences (Table 1).

In relation to depression, there was a 30.2% prevalence of depressive symptoms among Brazilian older people and a statistically significant (p < 0.001) difference between the genders (23.8% for men and 34.4% for women). For Chilean people, we observed a significantly higher percentage among females (28.3%) compared to males (22.7%). We observed more Chilean women with ADL limitations (p < 0.001). The prevalence of IADL limitations was similar between the two countries, 29.2% for Brazil and 28.9% for Chile. There were significant differences (p < 0.001) between men (23.7% for Brazilian and 10.1% for Chilean) and women (33.0% for Brazilian and 24.1% for Chilean) (Table 1).

### Results of bivariate analysis

Overall, 35.5% and 52.1% of individuals reported good SRH, in Chile and Brazil, respectively. The distribution of SRH according to physical health, social structure, behavioural/attitudinal and psychosocial factors is shown in Table 2. Poor SRH responses were significantly more frequent among women (64.7% for Brazilian and 69.2% for Chilean) than men.

**Table 2:** **Descriptive characteristics of self-related health among Brazilians and Chileans older adults**

**Variables**	**Self-related health Chile (N = 1300)**			**Self-related health Brazil (N = 2052)**		
	**Poor**	**Good**	**p**	**Poor**	**Good**	**p**
	**(N = 839)**	**(N = 461)**		**(N = 982)**	**(N = 1070)**	
**Physical health**						
**Chronic Diseases**	**n (%)**	**n (%)**		**n (%)**	**n (%)**	
≥2	488 (61.3)	165 (37.7)	<0.001	670 (68.2)	429 (40.1)	<0.001
1	247 (31)	205 (46.8)		231 (23.5)	395 (36.9)	
0	61 (7.7)	68 (15.5)		81 (8.2)	246 (23.0)	
**ADL Limitation**						
Yes	74 (11.5)	33 (7.2)	<0.001	113 (11.5)	47 (4.4)	<0.001
No	618 (88.5)	426 (92.8)		869 (88.5)	1023 (95.6)	
**IADL Limitation**						
Yes	605 (36.4)	68 (14.9)	<0.001	349 (35.5)	251 (23.5)	<0.001
No	533 (63.6)	389 (85.1)		633 (64.5)	819 (76.5)	
**Cognitive Impairment**						
Yes	91 (10.9)	26 (5.6)	0.002	149 (15.2)	115 (10.7)	0.003
No	748 (89.1)	435 (94.4)		833 (84.8)	955 (89.3)	
**Social structure**						
**Age**						
<75 years old	545 (65.0)	313 (67.9)	0.285	669 (68.1)	758 (70.8)	0.195
≥75 years old	294 (35.0)	148 (32.1)		313 (31.9)	312 (29.2)	
**Sex**						
Masculine	258 (30.8)	188 (40.8)	<0.001	347 (35.3)	479 (44.8)	<0.001
Feminine	581 (69.2)	273 (59.2)		635 (64.7)	591 (55.2)	
**Marital Status**						
Married	282 (33.6)	169 (36.8)	0.439	487 (49.7)	597 (55.8)	0.004
Separate	156 (18.6)	92 (20)		66 (6.7)	88 (8.2)	
Widower	336 (40.1)	168 (36.6)		327 (33.4)	285 (26.7)	
Single	65 (7.8)	30 (6.5)		100 (10.2)	99 (9.3)	
**Years of Education**						
0	164 (19.6)	58 (12.6)	<0.001	295 (30.0)	284 (26.5)	<0.001
1-4	231 (27.5)	107 (23.2)		626 (63.7)	656 (61.3)	
5-7	243 (29)	122 (26.5)		44 (4.5)	86 (8.0)	
>8	201 (24)	174 (37.7)		17 (1.7)	44 (4.1)	
**Income**						
1° tertile	265 (35)	96 (23)	<0.001	697 (71.0)	660 (61.7)	<0.001
2° tertile	250 (33)	133 (32)		219 (22.3)	286 (26.7)	
3° tertile	243 (32)	187 (45)		66 (6.7)	124 (11.6)	
**Retired**						
Yes	269 (35.4)	128 (30.0)	<0.001	722 (73.5)	796 (74.4)	0.687
No	490 (64.6)	299 (70.0)		260 (26.5)	274 (25.6)	
**Household Arrangement**						
Living with spouse	184 (21.9)	99 (21.5)	0.654	479 (49.5)	586 (56.2)	0.010
Mixed arrangements	543 (64.7)	308 (66.8)		345 (35.6)	323 (31.0)	
Living alone	112 (13.4)	54 (11.7)		144 (14.9)	133 (12.8)	
**Healthcare**						
Public	719 (59.4)	369 (53.3)	<0.001	583 (59.4)	570 (53.3)	0.003
Others	28 (3.3)	35 (7.6)		399 (40.6)	500 (46.7)	
**Behavioural/attitudinal**						
**Currently Smoking**						
Yes	94 (11.2)	64 (13.9)	0.153	116 (11.8)	109 (10.2)	0.258
No	743 (88.8)	395 (86.1)		866 (88.2)	961 (89.8)	
**Drinking**						
Yes	46 (5.5)	46 (10.0)	0.002	171 (17.4)	212 (19.8)	0.174
No	793 (94.5)	412 (90.0)		811 (82.6)	858 (80.2)	
**Physical Activity**						
Yes	150 (17.9)	120 (26.1)	<0.001	208 (21.2)	337 (31.5)	<0.001
No	688 (82.1)	339 (73.9)		774 (78.8)	733 (68.5)	
**Psychosocial**						
**Symptoms of Depression**						
Yes	207 (28.2)	98 (23.1)	0.055	430 (43.8)	189 (17.7)	<0.001
No	527 (71.8)	327 (76.9)		552 (56.2)	881 (82.3)	
**Social Participation**						
Yes	248 (29.7)	160 (34.8)	0.058	82 (8.4)	75 (7.0)	0.280
No	588 (66.2)	300 (65.2)		900 (91.6)	995 (93.0)	

ADL: activities of daily living.

IADL: instrumental activities of daily living.

In Table 2, statistically significant differences for SRH were found in both countries related to chronic diseases, ADL limitation, IADL limitation, cognitive impairment, sex, education, income, healthcare and physical activity. The frequency of good SRH was 2.3 times higher among non-retired Chilean older people compared to retired (p < 0.001). In Brazilian older adults, household arrangement and marital status were associated with good SRH. Brazilian older adults without depression had better perception of health compared to those with depression (p < 0.001).

### Hierarchical model of predictors of SRH

We estimated the OR of good SRH in regression models. Brazilian and Chilean adults without symptoms of depression had 3.3 (OR = 3.3, CI95% = 2.5-4.0) and 1.4 (OR = 1.4, CI95% = 1.0-2.0) higher odds of good SRH, compared with symptoms of depression (Additional files 1 and 2).

Good SRH was significantly predicted by no ADL limitation (OR = 2.1; CI95% = 1.0-4.3), no IADL limitation (OR = 1.8; CI95% = 1.1-3.0), <75 years old (OR = 1.6; CI95% = 1.1-2.3), 8 years of education (OR = 1.7; CI95% = 1.0-2.8), and healthcare (OR = 2.1; CI95% = 1.1-4.2). Older people with one or two chronic disease(s) had 3.6 and 2.2 higher chance of good SRH, compared to no chronic disease (Additional file 1).

There was an inverse gradient for association between good SRH and chronic diseases in all models. When controlling for potential confounds, the associations were attenuated but remained statistically significant. One chronic disease (OR = 2.4, CI95% = 1.9-3.0) and, in particular, no chronic disease (OR = 4.1, CI95% = 3.0-5.5), were associated with an elevated chance of good SRH (Additional file 2).

No ADL limitation, no IADL limitation, 5–7 years of education and no smoking were also strongly associated with good SRH, while all other variables were comparable. By contrast, no physical activity was a risk factor for poorer health perception (Additional file 2). In model 1, Brazilian adults without cognitive impairment had 1.4 higher probability of having good SRH than with cognitive impairment (p = 0.031). However, this association did not remain in the final model (Additional file 2).

### Gender analysis

Table 3 presents the results of significant associations between perceived health and predictors for females and males separately. There was a gradient association between good SRH and chronic diseases in both countries. Chilean men without chronic disease or with one had 3.3 and 2.9 higher chance of good SRH, compared to those with two or more diseases. For Brazilian men, no chronic disease (OR = 4.4, CI95% = 2.9-6.8) and one (OR = 2.6, CI95% = 1.8-3.6) were associated with good SRH (Table 3).

**Table 3:** **Final model predicting good self-related health in older adults separated by gender**

	**Chile** ^**a**^				**Brazil** ^**b**^			
	**Male**		**Female**		**Male**		**Female**	
	**OR**	**95% CI**	**OR**	**95% CI**	**OR**	**95% CI**	**OR**	**95% CI**
**Chronic Diseases**								
0	3.3**	1.9-5.7	4.4**	2.2-8.9	4.4**	2.9-6.8	3.9**	2.6-6.0
1	2.9*	1.3-6.0	2.1**	1.4-3.2	2.6**	1.8-3.6	2.2**	1.7-2.9
≥2	1.0		1.0		1.0		1.0	
**ADL Limitation**								
No	1.0	0.2-5.4	2.5*	1.1-5.5	1.1	0.6-2.0	2.5*	1.4-5.0
Yes	1.0		1.0		1.0		1.0	
**IADL Limitation**								
No	3.0*	1.0-9.0	1.2	0.7-2.4	1.5*	1.1-2.2	1.0	0.8-1.4
Yes	1.0		1.0		1.0		1.0	
**Physical Activity**								
No	0.7	0.4-1.2	0.8	0.5-1.2	0.7*	0.5-1.0	0.8	0.6-1.1
Yes	1.0		1.0		1.0		1.0	
**Depression**								
No	1.0	0.6-1.7	1.5	0.9-2.3	2.8**	2.0-4.1	3.1**	2.4-4.1
Yes	1.0		1.0		1.0		1.0	

^a^Controlling for age, education, income, retirement, healthcare, alcohol.

^b^Controlling for education, smoking.

*p < 0.05.

**p < 0.001.

For women, the set of independent predictors of good SRH included no chronic diseases (OR = 3.9 for Brazilian and OR = 4.4 for Chilean) or one chronic (OR = 2.2 for Brazilian and OR = 2.1 for Chilean) disease, and no ADL limitation (OR = 2.5 for both) (Table 3).

For men, the set also included no IADL limitation. The influence of functional limitation in health perception was 1.5 higher among Chileans adults (OR = 3.0, CI95% = 1.0-9.0) than Brazilian adults (OR = 1.5, CI95% = 1.1-2.2) (Table 3). Brazilian men who did not exercise had 70% higher probability of having poor SRH than men with physical activity (Table 3). For Brazilian adults of both genders, depression demonstrated the strongest independent association with good SRH. Men and women without depression had almost three times more chance of having good SRH (OR = 2.8 and OR = 3.1, respectively), compared to those with depression (Table 3).

## Discussion

This is one of the few studies from South American countries to study the effects of socioeconomic status and health on SRH among older adults when attempting to understand health inequalities between men and women.

Good SRH was 1.5 times higher among Brazilian older adults (52.1%) compared to Chileans (35.5%). Data from the SABE study indicated poorer health among older people of both sexes in the cities of São Paulo, Santiago and Mexico - countries with high levels of income inequalities [32]. A systematic review of SRH in Brazilian older people showed a prevalence of negative SRH between 12.6% and 51.9% [33].

These differences between the studies may have two possible explanations. The first is the absence of standardised questions and response options regarding SRH. In a study conducted in Armenia, the categories of response were “excellent”, “very good”, “good”, “fair” and “poor” which were dichotomised as fair/poor versus good [21]. In other studies, the authors chose to analyse very poor, poor and fair together versus very good/good [19,32]. These categorisations may overestimate the prevalence of negative or positive SRH. Secondly, comparisons are difficult given the different definitions used, as no reference values have been defined in the literature as acceptable for SRH among older people [33].

Women had worse health perception compared to older men in Brazil and Chile. These results were in agreement with previous studies conducted in Latin American and the Caribbean [24,32,34], and in others countries [21,35]. On other hand, this study contradicted other studies demonstrating better perception of health among women [19,36].

SRH has been shown to be a reliable method for measuring gender differences in health status [37] and a good predictor of mortality [38,39] among older people. However, in our study, multivariate analyses showed no independent association between gender and SRH in Brazil or in Chile.

The influence of gender in relation to older people’s health and SRH was confirmed in other studies [15,35] and further investigation is needed. This study provided evidence in the model separated by gender that SRH differed between men and women and varied between Brazil and Chile.

There was a consistent inverse relationship between chronic disease and SRH among Brazilians and Chileans older people. More interesting is that these differences remained when other variables were included in the model and when the results were separated by gender. Brazilian men and Chilean women seemed to report the highest SRH in the absence of chronic health, even after adjusting for confounds. Data from São Paulo in SABE indicated that in the absence of chronic diseases, or in the presence of two or more chronic diseases, older women self-assessed their health relatively better than older men [19].

This difference regarding the effect of gender on SRH countries may be related to differences in the sex ratio or in both samples. In Chile, there were 1.9 women for every man while the ratio for the sample in Brazil was 1.5. In addition, these data referred to both baseline studies. Therefore, it is not possible to determine whether these gender differences are cultural or whether there is a temporal relationship between SRH and gender and other variables.

In both countries, we observed the strongest association between ADL and IADL limitation with SRH, controlling for chronic diseases and cognitive impairment. In model 4, these associations were attenuated but remained statistically significant. Good SRH can be considered a protective factor against functional limitations [19,24,40].

Among women, no ADL limitation was statistically associated with good SRH. A majority of women who reported good health had no ADL limitation in Brazil (95.6%) and Chile (92.8%). Among men, no IADL limitation was statistically associated with good SRH. The chance among Chilean older people (OR = 1.5; CI95% = 1.1-2.2) was twice as high than among Brazilians (OR = 3.0; CI95% = 1.0-9.0).

The prevalence of functional limitations in older people was high, particularly among women [8,12,41]. While in Chile the prevalence of ADL limitation was higher among women, in Brazil there was an inverse relationship between the genders. Regarding IADL, the prevalence of limitation was higher among women compared to men in both countries.

Overall prevalence of depression was higher in Brazilian older people (30.2%) that Chilean older people (26.3%), but associations between SRH and depression were only found in Brazil . The chance of older people without depression reporting better health was slightly higher among women. This could be explained by the high prevalence of depression in women (34.4%), and, in particular, among women with poor SRH (72.0%). A study conducted in Spain showed the strongest relation to poor SRH with depressive symptoms (OR = 5.0), with important differences between genders (women, OR = 4.7 and men, OR = 5.2) [14].

Depression among women is a complex phenomenon that deserves to be investigated in relation to other health conditions and age [42]. We suggest that future studies can incorporate variables such as menopause, anxiety and clinical measures of women’s health between each age group (60–64 years, 65–69 years, 70–74 years and >75 years). We believe that considering gender differences among the ageing may contribute to the improvement of SRH and, ultimately, the welfare of this group.

### Limitations

SRH is among the most frequently assessed health perceptions in epidemiological research. However it is necessary to discuss some options for standardising questions and answers in order to follow and compare results to guide decisions about the best health policies for Latin America and the world [33]. In a study comparing different measures of SRH with respect to differences in age and sex groups, the authors concluded that non-comparative measures were more appropriate in longitudinal studies and that measures without specified response options might be less suitable for a study with older people [43].

In this study, we did not find any association between socioeconomic status, gender and SRH. The socioeconomic factors discussed in this study were limited to income and education. Furthermore, categorised income tertiles were measured in different currencies which were converted to U.S. dollars to compare the results. It is necessary to include other contextual (consumer goods, purchasing power) and cultural (local habits and customs) characteristics. Future research should also focus on the health effects of gendered differences in domestic and paid work, and on home and family roles as well as the interaction among gender, household crowding, and health [44].

## Conclusions

In conclusion, when examining gender differences in predictors of SRH, the similarities are greater than the differences between Brazilian and Chilean older people. In both countries, physical health was the most important predictor of SRH. In addition, the absence of depression was the strongest predictor of good health in Brazilian older people.

## Additional files

Additional file 1:
**Logistic Regression results for physical health, social structure, behavioural/attitudinal and psychosocial variables predicting good self-related health in Chilean older adults.**
Additional file 2:
**Logistic regression results for physical health, social structure, behavioural/attitudinal and psychosocial variables predicting good self-related health in Brazilian older adults.**
